# Value of Information Based Data Retrieval in UWSNs

**DOI:** 10.3390/s18103414

**Published:** 2018-10-11

**Authors:** Fahad Ahmad Khan, Sehar Butt, Saad Ahmad Khan, Ladislau Bölöni, Damla Turgut

**Affiliations:** 1 Department of Electrical Engineering and Computer Science, University of Central Florida, Orlando, FL 32816, USA; skhan@eecs.ucf.edu (S.A.K.); lboloni@cs.ucf.edu (L.B.); 2 Department of Electrical Engineering and Computer Science, University of Engineering & Technology Lahore, Punjab 54890, Pakistan; seharbutt@knights.ucf.edu

**Keywords:** under water sensor network, mobility, path planning, routing, quality of information, value of information, mobile sink, data mule, UWSN, AUV, QoS, QoI, VoI

## Abstract

Sensor nodes in underwater sensor networks may acquire data at a higher rate than their ability to communicate over underwater acoustic channels. Autonomous underwater vehicles may mitigate this mismatch by offloading high volumes of data from the sensor nodes and ferrying them to the sink. Such a mode of data transfer results in high latency. Occasionally, these networks need to report high priority events such as catastrophes or intrusions. In such a scenario the expectation is to have a minimal end-to-end delay for event reporting. Considering this, underwater vehicles should schedule their visits to the sensor nodes in a manner that aids efficient reporting of high-priority events. We propose the use of the Value of Information metric in order to improve the reporting of events in an underwater sensor network. The proposed approach classifies the recorded data in terms of its value and priority. The classified data is transmitted using a combination of acoustic and optical channels. We perform experiments with a binary event model, i.e., we classify the events into high-priority and low-priority events. We explore a couple of different path planning strategies for the autonomous underwater vehicle. Our results show that scheduling visits to sensor nodes, based on algorithms that address the value of information, improves the timely reporting of high priority data and enables the accumulation of larger value of information.

## 1. Introduction

Monitoring in underwater sensor networks (UWSNs) has emerged as a subject of active research with applications such as maritime security operations, infrastructure surveillance and maintenance, sea-life monitoring and sea pollutant and contaminant measurements [1,2]. Routing techniques in UWSNs differ from traditional sensor networks [1,2]. The physical deployment and maintenance of the nodes are difficult and underwater sensing abilities can be hindered by cloudy water and debris. Radio waves travel only very short distances under water. This is because water tends to absorb a large part of the electromagnetic spectrum. This limitation can be mitigated by using an acoustic communication medium. Acoustic signals can travel long distances underwater but they have relatively high latency and low bandwidth. Such bandwidth maybe sufficient for transmitting compressed data [3] but is not appropriate for large volumes of data such as video recordings.

As an alternative, the research community has proposed the use of mobile sinks to gather and offload the data in wireless multimedia networks [4,5]. In the case of UWSNs, an autonomous underwater vehicle (AUV) can be used as a mobile sink [6,7,8,9,10]. In such a scenario, the acoustic channel can be used for transmitting messages such as signaling information [11]. While an optical communication channel can be used to offload large quantities of data from the sensor nodes to AUVs which are in close proximity [11].

**Figure 1:**
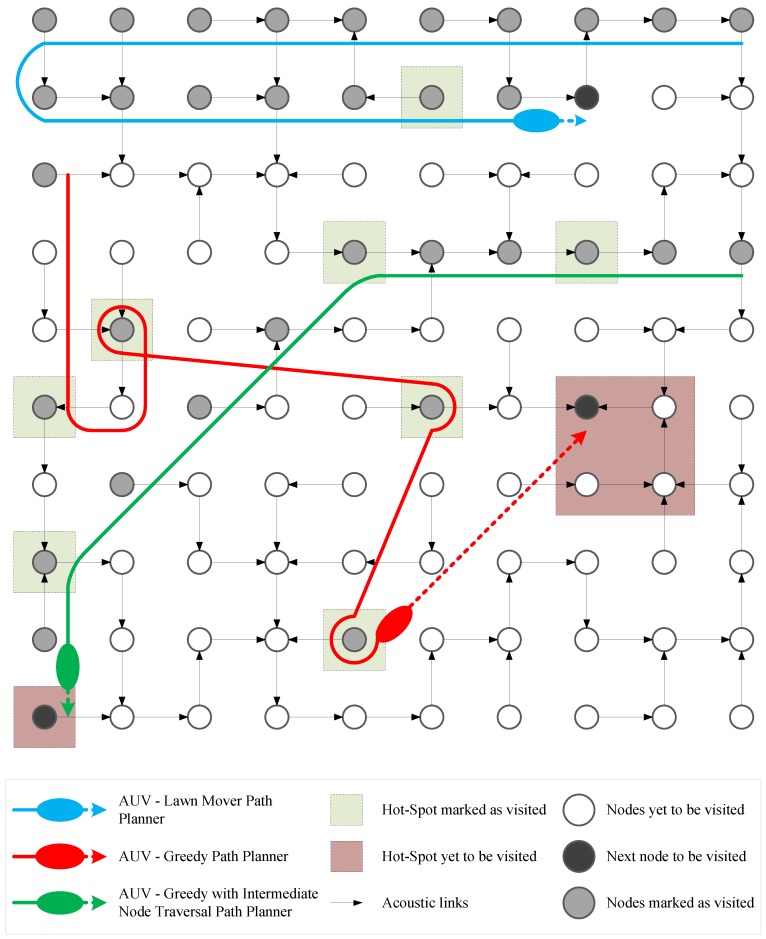
The UWSN mesh setting used for the simulation study. The same arrangement will be used for simulation and experimentation in the results section. The blue colored path is of the AUV that uses LPP, red of the AUV that uses GPP and green of the AUV that uses GIPP. The GPP visits one hot-spot after another. Notice how LPP stumbles upon a hot-spot, i.e., discovers it by chance. Also, observe how GIPP visits intermediate nodes in-between hot-spot visits.

An important issue for offloading data from sensor nodes using AUVs is the path planning for the AUV, i.e., the sequence in which the AUV visits the nodes for data offloading. An efficiently planned path can reduce the overall data delivery latency and increase the information accumulated over a set period of time. The visitation sequence also determines the order of priority in which emergency reporting nodes are catered to. Let us assume that there is a surveillance or monitoring application that must report a significant event on an urgent basis. In a traditional sensor network, the information can be sent to the sink node instantaneously by initiating a data transfer from either the sink or the source nodes. However, for UWSNs with mobile sinks, the sensor nodes must signal the event to a remote agent that controls the AUV. The AUV must travel to the required node for data collection. The tour time of an AUV depends upon the speed of the AUV and the weather and oceanic conditions. The timing of delivery of data from the sensor nodes to the sink is determined by the schedule of resurfacing, diving and node visitation. Therefore, the time taken to report the information about an event depends upon the choices made by the path planning algorithm for the AUV. Figure 1 describes a generalized example of such a monitoring application where an AUV has been deployed to retrieve data from the underwater sensor nodes.

In this paper, we extend our work regarding the use of the value of information (VoI) in UWSNs [12]. We employ VoI in a similar spirit to as it has been described in [13,14,15]. We argue the advantage of employing VoI in UWSNs through an example. We propose the use of VoI functions, which we have named as Infotentials. Infotentials are monotonically decreasing functions that model the temporal depreciation of VoI. Drawing from intuition based on these functions, we develop heuristics that are to be used for the AUV path planning. We formulate five different path planning algorithms for the AUVs for data collection purposes. In the end, we provide a detailed comparative analysis of the path planners for VoI maximization.

This paper has been organized in the following manner. Section 2 provides a literature survey of the usage of mobile sink nodes in underwater sensor networks, routing schemes involved in path planning and previous research work involving VoI. In Section 3.1 we introduce the concept of VoI for our scenario. Section 3.3 describes the UWSN setting that we are considering for our theory and experiments. Section 3.4 formulates the AUV path planning problem for the given UWSN setting. Section 4 outlines the path planning algorithms. Section 5.2 describes the setup of our simulation experiments. The results and conclusion are discussed in Section 5.3 and Section 6 respectively.

## 2. Related Work

### 2.1. Relationship between QoS, QoI, VoI and Routing

The over-arching goal of this article is to improve the QoI (quality of information) that can be obtained from a UWSN. In terms of the performance objectives, QoI is to sensor networks what QoS (quality of service) is to conventional computer and wireless networks [13,14,15]; although, technically speaking, in terms of implementation, QoI is built upon a QoS layer in sensor networks [16,17]. It is shown in [13,15] that the construct of QoI is intrinsically related to VoI, therefore, we use VoI for path planning purposes so as to improve QoI garnered from the system. Design of good routing algorithms is one of the fundamental aspects that dictates QoS and QoI in conventional networks [18], sensor networks [17,19,20,21,22] and networks with mobile elements [23,24]. Routing in networks is tailored to meet certain application-level or operational objectives. With the advent of IoT [25] and Fog-computing [26] paradigms, there continues to remain a keen interest in the development of routing algorithms to meet the new emergent objectives and constraints [27,28]. In this article, we employ a mobile sink (AUV) as part of the routing effort to deliver a better measure of VoI and hence QoI.

### 2.2. Some Applications of IQ and VoI in Sensor Networks

IQ (information quality) aware routing schemes make data routing decisions based on the threshold of information required by application or mission objectives [29,30,31]. The data is fused en-route to the sink node and routed on paths which can potentially satisfy the aggregated IQ threshold or constraints. Data is kept fusing incrementally on each next forwarding node until it reaches the IQ threshold. When the threshold is met, the information is sent to the main fusion center or sink node.

We use VoI in an event driven context. An event-based information quality aware routing (IQAR) has been proposed in [32]. The unique aspect about IQAR is its use of event-based data fusion, whereas, in the previous IQ aware routing algorithms, the fusion process would be initiated from the fusion centers. In the initial phase of the IQAR method, an aggregation tree is constructed that spans the whole network. In the case of an event, the sensors in vicinity forward the data using the preexisting links as established during the tree initialization. After data reaches the fusion center, the network uses a greedy approach to prune the initial tree in order to maximize the information retrieved and to minimize the energy consumption of the nodes on the data forwarding links.

One of the applications where IQ and VoI have been used in sensor networks is for tracking [30,33]. In [33] a VoI-based approach has been proposed for intruder tracking objectives. The sensor network, based on predictive measures centered around VoI, makes data routing decisions in the network for more efficient target tracking. The network is able to prioritize among high and low-value targets. Tracking a higher value target should result in a higher amount of VoI accumulated. In [34], the authors further proposed IVE which is an improved version of the VoI-based intruder tracking system. The network is able to control the demand of data packets while balancing trade-offs between network energy consumption and required VoI.

Concepts of quality and utility have been frequently applied to a variety of scheduling activities in sensor networks. Some examples of scheduling algorithms and protocols, which employ concepts of quality based on priority and utility metrics, are [35,36,37,38,39,40,41].

### 2.3. Using Mobile Sinks in Underwater Sensor Networks

Mobile sinks have been used as data mules in both sensor networks [5,42,43,44,45] and UWSNs [2,4,6,7,10]. Mobile sinks in UWSNs have been used in the form of AUVs [6,7] as well as dolphins [46]. In [46] it has been proposed to use a DDD (delay-tolerant data dolphin) to harvest the data from sensor nodes in the region of interest. The data collection event can be triggered by both the DDD or the sensor node. The DDD uses a bi-directional acoustic communication medium. As the movement of DDDs is random, therefore, there is a randomness associated with the event data collection, and this inhibits optimal performance.

VoI-based transmission scheduling of sensor nodes via acoustic links to the sink has been explored in [47]. While VoI-based path planning of an AUV to collect data from sensor nodes has also been explored in [12,48,49,50].

### 2.4. AUV Path Planning

Path planning is a diverse subject and there is a lot of literature on it specifically in the artificial intelligence community [51,52]. In terms of route discovery, a path planner is an algorithm that provides a sequence of steps which give a valid route between two points on a map (usually represented by a graph or a grid). Among the sub-goals or constraints of path planners are to find the most optimal route in terms of shortest distance, minimal time, optimal fuel expenditure, and so on. They essentially convert a set of high-level goal specifications into a sequence of low-level instructions, thus breaking down the problem solution into simpler steps.

Depending upon the nature of the environment, path planners can be static or dynamic based on how the information is being updated. Path planners, therefore, can also be classified as pre-computing algorithms or reactive algorithms [52]. If the path is planned before the mobile agent starts the course then the algorithm is pre-computing, while if the plan is updated during the course, in reaction to changes in objectives, constraints or new obstacles, then the algorithm is deemed reactive. Maps represented by graphs are usually solved by planners that are variants of Dijkstra’s Algorithm. One of the well-known variants is $A^{*}$ path planning which uses admissible heuristics to accelerate the path planning. The path planned in [12] can be seen as a two-tiered approach; the higher level algorithms, such LPP or GPP, determine the sequence of node visitation on the map, while $A^{*}$ provides the detailed sequence of steps required to travel from a source to a destination node.

AUVs are autonomous devices and hence require path planning techniques to help them navigate beneath seas and oceans. In terms of navigation, they have certain issues that hamper them more as compared to dry-land autonomous agents, which include communication limitations, limited sensing, and power issues. AUV path planning has been around for a while now. One of the earlier path planning techniques used for AUVs is case-based reasoning [53]. One of the first efforts to use A∗ for path planning for AUVs is in [54]. $FM^{*}$ is developed in [55] which gives a continuous path based on a discrete representation of the environment and also takes water currents into account. The path planning approach in [56] uses methods based on potential field strategies. This approach has been further improved on in [57]. Genetic Algorithms [58,59,60] and evolutionary algorithms [61] have also been employed for AUV path planning. Path planning for data mules has also been explored from the perspective of sensor networks in [44,45]. We have proposed path planning techniques for AUVs in UWSNs [12,49,50].

## 3. Value of Information in Underwater Sensor Networks

### 3.1. Value of Information

Assume there is a situation of interest that is being monitored so that it can be kept under control. Information is knowledge that helps in developing and updating a model of such a situation. It is based on this model that certain steps are taken to keep the situation in check. Value of Information (VoI) is the valuation of an information segment that is relevant to and can be added to this model.

Consider a marine environment that is being monitored by an underwater sensor network for reporting any catastrophic event. An example could be an offshore oil-rig and pipeline system that is under surveillance for events such as potential oil spills. The sensor nodes have video cameras which record information that can be processed to ascertain aging, rust, accidental damage or oil leaks. The higher the increase in the risk of an oil spill, as concluded from a model after adding an information segment, proportional is the increase in valuation of the information segment that leads to that conclusion.

From the perspective of multi-agent systems, VoI is the price a player would pay to acquire a piece of information in a game theoretic setting. This description is consistent with the usage of the term VoI in [62,63,64,65].

To have a more abstract view, let us consider a classic control theory scenario where, based on feedback, an agent has a certain degree of observability and controllability over a system. In other words, the agent can take specific actions in response to various observations so as to bring the state of the system to a rather more desirable one. The actions taken are such that some measure of fiscal profit or loss is incurred. These profits or losses are assessed from the current and future states of the system. The observations are part of the information based on which the future discourse of steps is decided by the agent. Therefore, information is a data report that can aid in building a more accurate model of a system such that the newer model aids in alleviating e.g., fiscal consequences.

### 3.2. Temporality of VoI; Infotentials

Infotential is a term that we have coined for information potential. By potential we imply the potential of impact an event has on an asset, and therefore, the report of such an event can be deemed to have a certain information potential. Infotentials are functions that encode the variability of VoI. VoI of a report is subject to various factors such as the valuation of the asset being reported; the impact of the event being reported on the asset; timeliness of the report; reliability, precision, and accuracy of the report; and the fact that whether this report is a repetition or not. Asset valuation dictates VoI but as there is a temporal dynamism to this valuation, therefore, VoI should correlate with this variability across time. We quote two factors that result in asset valuation decay; the first is that assets naturally depreciate with time; the other is that events of catastrophic nature can result in a sharper decline in the valuation of an asset. As both these fiscal attributes are decaying in nature, therefore, VoI should be a monotonically decreasing function.

In the context of this paper, we define a function that encodes the temporal variability of VoI. We consider this variability to be monotonically decreasing in time and assume that at any point in time the valuation of the asset does not increase due to any other factors. These functions can be designed in a variety of different ways so as to fit the needs of an application scenario. We model depreciation with a decaying exponential so as to maintain generality. Otherwise, functions such as descending staircases or any other complex combination of exponentials can also be used. Ideally, the function should be constructed based on practical statistical data.

In this study, we employ exponential functions of the form

(1) $$\upsilon \left(t\right)=Ae^{-B(t-\tau_{o})}$$

**Figure 2:**
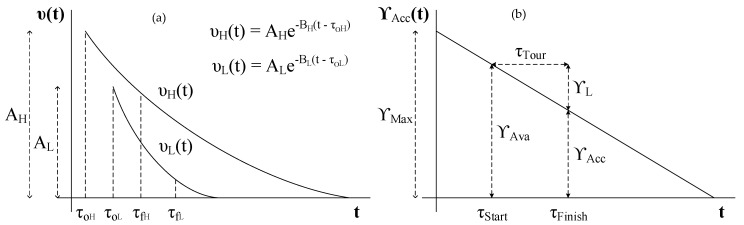
Infotentials: (**a**) The figure on the left is an example of exponentially decaying infotentials that we use for modelling and analyzing high-priority and low-priority events; (**b**) The figure on the right shows various parameters and measures that we use for comparative analysis of path planning algorithms.

Examples of the infotential υ(t) are shown in Figure 2a. The parameters that control the function are *A* and *B* and $\tau_{o}$. The parameter $\tau_{o}$ is the time at which the event is reported. Parameters *A* and *B* scale the VoI across the domain and range of the function. Parameter *A* represents the valuation of information at $t=\tau_{o}$ while parameter *B* represents the decay in information valuation *A* for $t>\tau_{o}$, i.e., after the onset of the event being reported at $\tau_{o}$. The two exponential functions in Figure 2a could be understood as representing two different classes of information in an application scenario. The more the valuation of an asset is, the higher the number *A* would be. Likewise, the higher the *B* value is, greater is the rate of damage to an asset.

Let there be two events $E_{H}$ and $E_{L}$ with significance and damage rate $\left\{S_{H},D_{H}\right\}$ and $\left\{S_{L},D_{L}\right\}$ respectively. The subscript *H* corresponds to a high-priority event while the subscript *L* indicates a low-priority event. We denote the corresponding VoI functions as,

(2) $$\begin{array}{cc} \upsilon_{H}\left(t\right) & =A_{H}e^{-B_{H}\left(t-\tau_{oH}\right)} \end{array}$$

(3) $$\begin{array}{cc} \upsilon_{L}\left(t\right) & =A_{L}e^{-B_{L}\left(t-\tau_{oL}\right)} \end{array}$$

The relationship between significance and valuation, and between damage rate and decay, is given as,

(4) $$\begin{array}{cc} S_{H}>S_{L} & \Rightarrow A_{H}>A_{L} \end{array}$$

(5) $$\begin{array}{cc} D_{H}>D_{L} & \Rightarrow B_{H}>B_{L} \end{array}$$

These inequalities are general guidelines. The actual values of parameters $A_{x}$ and $B_{x}$ need to be configured with the help of a system expert or should be based on statistical data.

### 3.3. UWSN Deployment Scenario

We consider a UWSN that has been deployed for monitoring the sea and its surface from below. This UWSN records video data. We assume that the UWSN has been deployed to monitor disasters such as oil spills and leaks from vessels or pipelines. Sensor nodes in the UWSN have the ability to detect and classify such disasters. Therefore, we have two classes of information reported; one is normal routine data while the other is data pertaining to disasters. Both classes of information are mapped to separate infotentials. The information for high-priority events such as oil leaks is mapped to the function $\upsilon_{H}\left(t\right)$ while normal routine information is classified as low-priority and is mapped to $\upsilon_{L}\left(t\right)$. The UWSN considered in our simulation study is illustrated in Figure 1. We assume a UWSN with *n* sensor nodes

(6) $$S=\{s_{1},s_{2},\ldots ,s_{i},\ldots ,s_{n}\}$$

These nodes are equipped with sensors that collect high-quality video data which makes their transmission over acoustic channels unfeasible. An AUV is deployed to gather data from these sensor nodes. The sensor nodes have acoustic communication modules for long distance communication such as transmitting signaling and configuration messages while optical communication modules are used for short distance transmissions such as offloading the data from the sensor nodes to the AUVs. The sensor nodes have the ability to classify data into *q* different information classes

(7) $$C=\{c_{1},c_{2},\ldots ,c_{p},\ldots ,c_{q}\}$$

Each information class is characterized by a unique infotential. The VoI functions that we use, as discussed earlier, are of the form

(8) $$\upsilon \left(t\right)=Ae^{-B(t-\tau_{o})}$$

In this case, the infotentials for the high-priority and low-priority information classes are as follows respectively

(9) $$\upsilon_{H}\left(t\right)=A_{H}e^{-B_{H}\left(t-\tau_{oH}\right)}$$

(10) $$\upsilon_{L}\left(t\right)=A_{L}e^{-B_{L}\left(t-\tau_{oL}\right)}$$

The sensor nodes process and save the data in the form of data reports. After time *t* the *i${}_{th}$* sensor node has *k* data reports

(11) $$D_{i}=\left\{d_{i1},d_{i2},\ldots ,d_{ij},\ldots ,d_{ik}\right\}$$

Data reports are mapped to specific information classes such that the mapping is surjective. This means that a classification function α (a computational procedure in the sensor node) may assign more than one data report to the same information class

(12) α:D→C

In terms of the *i*th sensor node and *j*th data report, the infotentials are denoted with subscripts as

(13) $$\upsilon_{ij}\left(t\right)=A_{ij}e^{-B_{ij}\left(t-\tau_{ij}\right)}$$

The information class identity is stored in a tag associated with each data report. This tag contains the necessary information required to reconstruct the infotential at the remote user’s end. The tag in our case is completely characterized by constants A, B and time stamp $\tau_{o}$ where $\tau_{o}$ is the time at which the data report was recorded by the sensor node. A tag is a 3-tuple entity. The tag for the *j*th data report at the *i*th sensor node is

(14) $$\lambda_{ij}=\left(A_{ij},B_{ij},\tau_{o_{ij}}\right)$$

Because the data reports are large in size, therefore, it is not possible to transmit them over the acoustic channel as stated earlier. The acoustic channel will be used for broadcasting the $\lambda_{ij}$ tags residing at a sensor node. The tags will be transmitted to a sink node. On the other hand, the optical channel will be used for transmitting the actual data reports to the AUV during its tour. The packets transmitted over the acoustic communication channel by the *i*th sensor node are composed of the payload $\lambda_{ij}$ (the VoI tag) and $\delta_{ij}$ (protocol, header information etc.). These packets are intended for delivery to a remote agent (sink node) and on the basis of this data, i.e., $\lambda_{ij}$ the sink node will plan a schedule of node visitation for the AUV. The packets are

(15) $$\Gamma_{i}^{Acoustic}=\left\{{\left(\lambda_{ij},\delta_{ij}\right)}_{1},\ldots ,{\left(\lambda_{ij},\delta_{ij}\right)}_{x}\right\}$$

The packets transmitted over the optical communication channel by the *i*th sensor node are composed of the $d_{ij}$ (the data report), $\lambda_{ij}$ (the VoI tag) and $\delta_{ij}$ (protocol, header information etc.). These packets have the actual recorded data of the events. The data is offloaded from the sensor nodes onto the AUV through this optical channel. The packets are

(16) $$\Gamma_{i}^{Optical}=\left\{{\left(d_{ij},\lambda_{ij},\delta_{ij}\right)}_{1},\ldots ,{\left(d_{ij},\lambda_{ij},\delta_{ij}\right)}_{y}\right\}$$

As soon as the data is received and processed at the remote user’s end, another timestamp $\tau_{f_{ij}}$ is assigned to the data report which helps in determining its current VoI from the infotential. This substitution yields:

(17) $$\upsilon_{ij}=A_{ij}e^{-B_{ij}\left(\tau_{f_{ij}}-\tau_{o_{ij}}\right)}$$

### 3.4. Problem Definition

The goal is to extract the maximum amount of VoI so as to minimize system and asset losses. To maximize the VoI retrieved, the path planners will need to determine a route that is efficient in terms of accumulating VoI from the system.

#### 3.4.1. VoI Maximization

We use the problem definition from our previous work in [50]. It is the same problem definition that has been used in [12,49] implicitly and [50] explicitly. The subtle difference, however, lies in the way the final time-stamp $\tau_{f}$ is determined. The VoI for a data report that is ready to be transmitted to the sink node is given as

(18) $$\upsilon_{ij}=A_{ij}e^{-B_{ij}\left(\tau_{f_{ij}}-\tau_{o_{ij}}\right)}$$

The aggregated VoI as retrieved from the *i${}_{th}$* sensor node is the combined VoI of all data reports residing on the sensor node and is given as

(19) $$\Upsilon_{i}\left(t\right)=\sum_{j=1}^{k}A_{ij}e^{-B_{ij}\left(t-\tau_{o_{ij}}\right)}$$

Similarly, the total VoI accumulated from the UWSN by the AUV, after it has visited all the sensor nodes based on a planned tour, can be calculated as

(20) $$\Upsilon_{Acc}\left(t\right)=\sum_{i=1}^{n}\Upsilon_{i}\left(t\right)=\sum_{i=1}^{n}\sum_{j=1}^{k}A_{ij}e^{-B_{ij}\left(t-\tau_{o_{ij}}\right)}$$

Therefore, the goal of maximizing VoI is defined as

(21) $$\max\sum_{i=1}^{n}\sum_{j=1}^{k}A_{ij}e^{-B_{ij}\left(t-\tau_{o_{ij}}\right)}$$

#### 3.4.2. Role of $\tau_{f}$ in Infotentials

The time-stamp $\tau_{f}$ determines the VoI extracted from an information segment based on its infotential. Therefore, VoI of a data report varies according to the time instant at which the information is gathered by an end-processing agent. In the given UWSN scenario, we have two different final time-stamp $\tau_{f}$ definitions. The definitions are based on who the end-processing agent is; the one who is responsible for triggering an actuation response after processing the data. If the AUV is equipped with the ability to process the data such that it can initiate an actuation response then $\tau_{f}$ is the time at which the AUV retrieves the data from the respective sensor node. However, if the end-processing agent is above the sea surface, as in [50], then $\tau_{f}$ is determined when the information is received by the end-processing agent (for which the AUV will have to resurface [50]). The time stamp $\tau_{f}$ definition can crucially impact the design of AUV path planning algorithms for VoI maximization. In this paper we assume the AUV to be the end-processing agent which implies that $\tau_{f}$ will be determined by instant at which the information segment was offloaded from the sensor node on to the AUV.

#### 3.4.3. The Path Planning Problem

The problem definition for AUV path planning is to devise an algorithm that attempts to maximize VoI accumulated from the UWSN. More formally, given sensor nodes *S* and VoI profile $\Upsilon_{Acc}\left(t\right)$ of data reports *D*; what is the sequence of node visitation $P_{S}$ in *S* that will result in the accumulation of VoI $\Upsilon_{Acc}^{Alg^{PP}}$.

(22) $$\begin{array}{c} \left[\Upsilon_{Acc}^{Alg^{PP}}\leftarrow P_{S}]\leftarrow Alg^{PP}[S,D,\Upsilon_{Acc}\left(t\right)\right] \end{array}$$

where, $\Upsilon_{Acc}^{Alg^{PP}}$ is the VoI accumulated from the sensor nodes *S* by employing the traversal sequence $P_{S}$,

$P_{S}$ is the node visitation sequence determined by $Alg^{PP}$,
$Alg^{PP}$ is a path planning algorithm that generates path $P_{S}$ such that $\Upsilon_{Acc}^{Alg^{PP}}$ is accumulated,
*S* is the set of all sensor nodes,
*D* is the set of all data reports,
$\Upsilon_{Acc}\left(t\right)$ is the cumulative VoI function.

## 4. Path Planning Algorithms

The path planning algorithms provide us with the sequence of nodes which the AUV will traverse for data collection. As suggested earlier, the sequence of node visitation by the AUV will affect the overall accumulated VoI. We propose a few different path planning algorithms for experimentation and analysis. The first algorithm is the Lawn-Mower path planner (LPP) which is based on minimizing total tour time. Then we have the Greedy path planner (GPP) which is based on greedily accumulating VoI. Greedy with Intermediate-Node-Visitation (GIPP) is a variant which is based on greedily accumulating VoI while also trying to minimize tour time. Hybrid path planner (HPP) is a combination of LPP and GPP. Hybrid with Intermediate-Node-Visitation (HIPP) is a combination of LPP and GIPP. The Random path planner (RPP) is used as a baseline algorithm for analysis purposes.

### 4.1. Lawn-Mower Path Planner–LPP

The LPP algorithm is based on a pre-computing strategy. It determines the route before-hand and it does not take into account the VoI profiles of the sensor nodes for its path planning decisions. The crux of this algorithm is to find the most optimal tour in terms of time traveled. This, in essence, is like solving the traveling salesman problem (TSP). The title Lawn-Mower is motivated by the analogy that the AUV will cover the area in parallel tracks in the same fashion as the grass is mowed down on a lawn using a lawn-mowing engine. A potential path planned using the LPP algorithm is shown with the blue colored trail in Figure 1.

Please note that this path planner does not explicitly take VoI into account but it should give effective results as it essentially takes into consideration the minimization of time *t*, which is a parameter in determining VoI as suggested by Equations (17), (19) and (20). Therefore, this algorithm implicitly improves VoI by taking care of overall visitation time.

Algorithm 1 highlights the steps involved for the Lawn-Mower path planner. The AUV maintains a history of nodes that it has already visited and the ones that it has yet to visit. Besides this, it also maintains a direction priority list. This list contains elements related to all the sensor nodes such that each element contains the information about which node among the neighbors of a sensor node needs to be visited next. For our implementation, we prioritize the selection of the next neighbor that is located on the East of the current node, then West, followed by South and finally North. The  complexity of LPP is O(n) where n is the number of sensor nodes.

A more generic implementation of this algorithm, for any topology other than a mesh, would be to use an algorithm that solves the TSP. However, for a mesh, instead of using a computationally expensive TSP algorithm, one can just simply use the LPP.

**Algorithm 1** Shortest Path Lawn-Mower Path Planner–**LPP**.1: **procedure**
LPP(*S*)2:     $S\leftarrow \{s_{1},s_{2},\ldots ,s_{n}\}$▹ Set of sensor nodes3:     V←∅
▹ Visitation sequence4:     $P_{D}\leftarrow \left\{\left(East,West,South,North\right)\right\}$
▹ Direction priority list5:     $i\leftarrow s_{1}$
▹ Tour starting node6:     **while**
S≠∅
**do**7:         N←
neighborhood(i,S)8:         $j\leftarrow s_{x}$ from *N* in the direction given by $P_{D}$9:         tourEculidean(i,j)10:         V←V+j11:         S←S−j12:         i←j13:     **end while**14:     **return**
*V*15: **end procedure**

### 4.2. Greedy Path Planner–GPP

GPP and its variants are reactive path planning algorithms. This path planner provides a visitation sequence of nodes in descending order of their VoI aggregate profiles, i.e., the AUV will visit the node with maximum VoI available first and then other nodes in decreasing order of VoI at offer by them. The VoI is determined as the value available at the time instant when the AUV visits the sensor node. This is a greedy approach as the algorithm tries to maximize the VoI accumulated by determining which node has the highest amount to offer and then visit it. A potential path planned using the GPP algorithm is shown with the red colored trail in Figure 1.

VoI accumulation is dependent on the final time-stamps $\tau_{f}$ as suggested by Equation (17). The lesser the value of $\tau_{f}$, the higher the VoI accumulated, i.e., the earlier the data is retrieved from a node, the more quickly the value decay is locked down, hence, resulting in greater value dividends. It would, therefore, make more sense to lock down VoI at nodes that have higher values to offer. Otherwise, more VoI would be potentially lost.

If there is a scenario where high-priority events are being reported because of a catastrophe, then it is imperative that it be reported at the earliest. The more the delay in reporting catastrophic data, the more would be the losses incurred. Moreover, as the UWSN has been deployed to report of catastrophic events, therefore, it is necessary that the AUV visits the nodes reporting the catastrophe first.

Algorithm 2 details the steps for the greedy approach. The algorithm maintains a history of nodes that it has already visited and the ones that it has yet to visit. From the nodes not visited yet, it selects the ones that give it the maximum VoI at its time of arrival. Once it selects a node as its next destination, it takes a direct euclidean route to it. The complexity of GPP is $O(n^{2})$ where n is the number of sensor nodes.

**Algorithm 2** Greedy Path Planner–**GPP**.1: **procedure**
GPP(*S*, $s_{o}$)2:     $S\leftarrow \{s_{1},s_{2},\ldots ,s_{n}\}$▹ Set of sensor nodes3:     V←∅
▹ Visitation sequence4:     $i\leftarrow s_{o}$
▹ Tour starting node5:     **while**
S≠∅
**do**6:         j←
getNodeThatHasMaxVoI(*S*)7:         tourEculidean(i,j)8:         V←V+j9:         S←S−j10:     **end while**11:     **return**
*V*12: **end procedure**13: **procedure**
getNodeThatHasMaxVoI($S_{r}$)14:     $\forall s_{x}\in S_{r}$ determine $\Upsilon_{s_{x}}$ using DetermineNodeVoI($D_{s_{x}}$, *t*)15:     $k\leftarrow s_{x}\in S_{r}$ such that $\Upsilon_{s_{x}}$ is $\max\sum Ae^{-B(t-\tau_{o})}$16:     **return**
*k*17: **end procedure**18: **procedure**
DetermineNodeVoI(D,t)19:     $D\leftarrow \{d_{1},d_{2},\ldots ,d_{k}\}$
▹ Data reports at node20:     Υ←021:     $\tau_{f}\leftarrow t$
▹ AUV arrival time at node22:     **while**
D≠∅
**do**23:         j←
GetNextDataReport(*D*)24:         α←
Get$A_{x}$(*j*)25:         β←
Get$B_{x}$(*j*)26:         $\tau_{o}\leftarrow$
Get$\tau_{o_{x}}$(*j*)27:         Υ+=
$\alpha e^{-\beta (\tau_{f}-\tau_{o})}$28:         D←D−j29:     **end while**30: **end procedure**

### 4.3. Greedy Path Planner with Intermediate-Node-Visitation–**GIPP**

It is a similar algorithm to GPP but with the added notion that while the AUV is on its way to the next node with the highest VoI offer, it can visit nodes that (by some definition e.g., distance) lie on the prescribed path.

It might be a reasonable idea to visit intermediate nodes, i.e., nodes that lie on the path to the destination node. This will help in minimizing tour time and, hence, improving VoI accumulation.

Algorithm 3 lays out the steps for GIPP. It is similar to GPP except for the intermediate node visitation which is a slight detour. For the intermediate node visitation, the algorithm selects the next node to be visited from the neighbors of the current node in a manner that the distance between the neighbor node and the node with the maximum VoI (destination node) is minimum among all the neighboring nodes. This algorithm is inspired by concepts behind both the GPP and LPP, i.e., visit sequence is greedy but tour time is minimized by visiting nodes that lie across the path. However, when it comes to reporting catastrophes at the earliest, it is a tad slower than GPP because of the intermediate node visitations. A potential path planned using the GIPP algorithm is depicted by the green colored trail in Figure 1.

The complexity of GIPP is $O(n^{2}+n\times d\times m)$ where *n* is the number of sensor nodes, *d* is the count of nodes in the longest intermediate tour, and *m* is the maximum number of nodes in a neighborhood definition. Because d<n and also m<n, therefore, it follows that the complexity of GIPP is $O(n^{3})$.

**Algorithm 3** Greedy Path Planner with Intermediate-Node-Visitation–**GIPP**.1: **procedure**
GIPP(*S*, $s_{o}$)2:     $S\leftarrow \{s_{1},s_{2},\ldots ,s_{n}\}$▹ Set of sensor nodes3:     V←∅
▹ Visitation sequence4:     $i\leftarrow s_{o}$
▹ Tour starting node5:     **while**
S≠∅
**do**6:         j←
getNodeThatHasMaxVoI(*S*)7:         T←∅8:         T←
tourIntermediate(i,j,S)9:         V←V+T10:         S←S−T11:         i←j12:     **end while**13:     **return**
*V*14: **end procedure**15: **procedure**
tourIntermediate($source,destination,S_{r}$)16:     p←source17:     q←destination18:     $T_{I}\leftarrow \emptyset$
▹ Intermediate Visitation Sequence19:     **while**
p≠q
**do**20:         N←
getNeighborhood($p,S_{r}$)21:         $i\leftarrow s_{x}\in N$ such that eculideanDistance($s_{x},q$) is minimized22:         tourEculidean(p,i)23:         p←i24:         $T_{I}\leftarrow p$25:     **end while**26:     **return**
$T_{I}$27: **end procedure**

### 4.4. Hybrid Path Planner–HPP

The algorithms for path planners HPP and HIPP were devised after studying results from our simulation experiments. What we observed was that the LPP would perform best, in terms of VoI accumulation, when there where no catastrophes, i.e., VoI profiles were similar across all the sensor nodes. Contrarily, GPP and GIPP performed better in the scenario where the sensor nodes were reporting catastrophes.

**Algorithm 4** Hybrid Path planner–**HPP**.1: **procedure**
HPP(*S*, $s_{o}$)2:     $S\leftarrow \{s_{1},s_{2},\ldots ,s_{n}\}$
▹ Set of sensor nodes3:     V←∅
▹ Visitation sequence4:     $S_{HP}\leftarrow \{s_{m}|s_{m}\in S\wedge s_{m}$ is high-priority} ▹ Set of high-priority sensor nodes5:     $S_{LP}\leftarrow S-S_{HP}$
▹ Set of low-priority sensor nodes6:     V←
GPP($S_{HP}$, $s_{o}$)7:     V←V+
LPP($S_{LP}$)8:     **return**
*V*9: **end procedure**

So the thought process behind this path planner is to use a greedy algorithm (GPP or GIPP) for retrieving data from nodes that are reporting catastrophes, while, using TSP like algorithms (LPP) for retrieving data from the rest of the nodes. This implies switching a greedy and a shortest path algorithm and so the name Hybrid Path Planner. HPP switches between GPP and LPP.

The algorithm for HPP is given as Algorithm 4. The complexity of this algorithm is $O(h^{2}+l)$ where *h* is the number of high-priority sensor nodes while *l* is the count of low-priority sensor nodes. $O(h^{2})$ is the complexity of GPP being used within HPP while O(l) is the complexity of LPP. As h≤n and l≤n, therefore, the complexity of HPP is determined as $O(n^{2})$.

### 4.5. Hybrid Path Planner with Intermediate-Node-Visitation–HIPP

HIPP has the same logic behind it as HPP but the only difference is that it uses GIPP instead of GPP. The algorithm first discovers the hot-spots, i.e., catastrophe reporting nodes and then it finds the intermediate nodes that can be visited. Afterward, these nodes are visited using GIPP while the rest of the nodes are visited using LPP.

The algorithm for HIPP is given as Algorithm 5. The complexity of this algorithm is $O(h^{3}+l)$ where *h* is the number of high-priority sensor nodes while *l* is the count of low-priority sensor nodes. Similarly, as in the case of HPP, h≤n and l≤n, therefore, the complexity of HIPP is determined as $O(n^{3})$.

**Algorithm 5** Hybrid Path Planner with Intermediate-Node-Visitation–**HIPP**.1: **procedure**
HIPP(*S*, $s_{o}$)2:     $S\leftarrow \{s_{1},s_{2},\ldots ,s_{n}\}$
▹ Set of sensor nodes3:     V←∅
▹ Visitation sequence4:     $S_{HP}\leftarrow \{s_{m}|s_{m}\in S\wedge s_{m}$ is high-priority } ▹ Set of high-priority sensor nodes5:     $i\leftarrow s_{o}$
▹ Sensor node to start tour from6:     **while**
$S_{HP}\neq \emptyset$
**do**7:         j←
getNodeMaxVoI($S_{HP}$)8:         T←∅9:         T←
tourIntermediate(i,j,S)10:         V←V+T11:         $S_{HP}\leftarrow S_{HP}-T$12:         i←j13:     **end while**14:     $S_{LP}\leftarrow S-V$
▹ Set low-priority sensor nodes15:     V← V + LPP($S_{LP}$)16:     **return**
*V*17: **end procedure**

### 4.6. Random Path Planner–RPP

For evaluation purposes, we also implemented a random path planner (RPP) where the AUV randomly chooses the next sensor node for data collection. The route towards the selected node employs Euclidean shortest path. This path planner can be thought of as a planner which does not take into account VoI or time while scheduling visits to nodes, i.e., AUV schedules its visitation activity irrespective of the critical nature of the VoI in the UWSN.

## 5. Performance Measures, Simulation Setup and Results

### 5.1. Performance Measures and Experiments

The basic measures of performance that we will use are VoI accumulated ‘$\Upsilon_{Acc}$’ and VoI lost ‘$\Upsilon_{L}$’. To discuss what these measures are we refer to Figure 2b. $\Upsilon_{Acc}\left(t\right)$ is the decaying VoI profile in the system and is represented in abstract terms by a straight line. It is a general statement on the depreciation of the valuation, whereas, in an actual situation the dynamics of this depreciation, i.e., the actual shape of the curve, will be governed by system variables and type of information recorded. This chart assumes that no measurements are recorded after t=0, hence, the chart only shows a monotonic decay in the valuation after t=0. $\tau_{start}$ and $\tau_{finish}$ are the start and end times for the complete AUV tour. In our case, a tour is a visitation sequence that is a permutation on the set of all sensor nodes, i.e., the tour is a ’simple path’ on a graph that has the sensor nodes as vertices. $\Upsilon_{Ava}$ is the VoI available in the system at the start of the tour while the $\Upsilon_{Acc}$ is the VoI accumulated by the AUV by the end of its tour. $\Upsilon_{L}$ is determined as

(23) $$\begin{array}{c} \Upsilon_{L}=\Upsilon_{Ava}-\Upsilon_{Acc} \end{array}$$

(24) $$\Upsilon_{L}=\sum_{i=1}^{n}\sum_{j=1}^{k}\left[A_{ij}e^{-B_{ij}\left(\tau_{Start}-\tau_{o_{ij}}\right)}-A_{ij}e^{-B_{ij}\left(\tau_{f_{ij}}-\tau_{o_{ij}}\right)}\right]$$

The loss in $\Upsilon_{Acc}$ is a result of a combination of factors that can be attributed to physical system limitations such as the AUV speed, delay in starting time of the tour $\tau_{Start}$, and inefficiencies resulting from the planned path. It is the path planning part of this problem that we want to explore in this paper. To filter out effects of a delay in $\tau_{Start}$ we use $\Upsilon_{L}$ as it is a measure of loss between the range $t=[\tau_{Start}:\tau_{Finish}]$. Any loss other than $\Upsilon_{L}$ is not a result of path planning inefficiencies. $\Upsilon_{L}$ gives a more clear picture in terms of comparative performance as compared to $\Upsilon_{Acc}$.

Other than VoI based performance markers we can also use time as a metric to determine certain aspects of performance. Time taken by an AUV for a complete traversal of the set of sensor nodes can be employed for this purpose. The earlier an AUV completes the tour of a UWSN, the more readily it is available for a new tour of this or another neighboring UWSN. Time can be useful in understanding energy $E_{AUV}$ consumed by the AUV to complete its tour. Energy consumed is proportional to the distance it travels $D_{tour}$ which is proportional to the time taken by the AUV to complete the tour $T_{tour}$. Shorter tours also result in less wear and tear of the AUVs.

(25) $$\begin{array}{c} \Delta \tau =\tau_{finish}-\tau_{start}=T_{tour}\propto D_{tour}\propto E_{AUV} \end{array}$$

A measure of the efficiency Ω of a planner can be determined by combining $\Upsilon_{L}$ and $T_{tour}$. Ω is inversely proportional to both $\Upsilon_{L}$ and $T_{tour}$, therefore,

(26) $$\begin{array}{c} \Omega \propto \frac{1}{\Upsilon_{L}}\cdot \frac{1}{T_{tour}}\Rightarrow \Omega =\frac{p}{q\Upsilon_{L}\cdot rT_{tour}}=\frac{k}{\Upsilon_{L}\cdot T_{tour}} \end{array}$$

where, $k=\frac{p}{q\cdot r}$ is a constant and is set to 1 in this experimental study. Other than the measures of $\Upsilon_{Acc}$, $\Upsilon_{L}$, $T_{tour}$ & Ω, we also need to have some specific measures for ’response to emergency situations’. These would be VoI accumulated from first hot-spot $\upsilon_{HS_{Acc}}$, VoI lost from first hot-spot $\upsilon_{HS_{L}}$, time taken to arrive at first hot-spot $\tau_{HS}$ and a measure of urgency Ψ. We define Ψ as the ratio of the score of the path planner schedule for visiting hot-spots $S_{I}$ to the score of an expected perfect schedule to visit hot-spots $S_{P}$. Let there be *n* sensor nodes and *m* hot-spots. Then, the score $S_{I}$ & $S_{P}$ are determined as

(27) $$\begin{array}{c} S_{I}=\sum_{i=0}^{m-1}n-seq{\#}_{s_{i}},S_{P}=\sum_{i=1}^{m-1}n-i \end{array}$$

where $seq{\#}_{s_{i}}$ is the number at which a node is visited in the schedule of visitation given by a path planner. Ψ is, therefore, determined as

(28) $$\begin{array}{c} \Psi =\frac{S_{I}}{S_{P}}=\frac{\sum_{i=0}^{m-1}n-seq{\#}_{s_{i}}}{\sum_{i=1}^{m-1}n-i} \end{array}$$

The intuition behind urgency score Ψ can be understood by the following example. Assume that there is a sensor network with 8 sensor nodes $\left\{s_{1},s_{2},s_{3},s_{4},s_{5},s_{6},s_{7},s_{8}\right\}$ and 3 hot-spots $\left\{s_{3},s_{6},s_{7}\right\}$. Here n=8 & m=3. The hot-spots in sequence of their precedence are $\left[s_{6},s_{3},s_{7}\right]$, i.e., it is most urgent to visit $s_{6}$ on priority, then $s_{3}$ and then $s_{7}$. The perfect visitation sequence should be $\left[s_{6},s_{3},s_{7},\ldots \right]$. Let there be a path-planner PP such that it gives us the following schedule of visitation $\left[s_{1},s_{3},s_{2},s_{6},s_{5},s_{7},s_{8},s_{4}\right]$. In this visitation sequence $s_{6}$ has a $seq{\#}_{s_{6}}=3$, $s_{3}$ has $seq{\#}_{s_{3}}=1$ while $s_{7}$ has $seq{\#}_{s_{7}}=5$. In a perfect visitation sequence $s_{6}$ should have $seq{\#}_{s_{6}}=0$, $s_{3}$ should have $seq{\#}_{s_{3}}=1$ while $s_{7}$ should have $seq{\#}_{s_{7}}=2$. We can now calculate $S_{I}$ and $S_{P}$ as $S_{I}=\left(8-3\right)+\left(8-1\right)+\left(8-5\right)=15$ and $S_{P}=\left(8-0\right)+\left(8-1\right)+\left(8-2\right)=21$. The smaller a $seq{\#}_{s_{i}}$ is, the higher the difference $n-seq{\#}_{s_{i}}$ will be and hence the greater the score. The urgency score for PP is $\Psi =S_{I}/S_{P}=15/21=0.714$.

Therefore, the complete list of measures that we use in this study for adjudication are $\Upsilon_{ACC}$, $\Upsilon_{L}$, $T_{tour}$, Ω, $\upsilon_{HS_{L}}$, $\tau_{HS}$ & Ψ.

### 5.2. Simulation Setup

To investigate our various hypotheses regarding VoI based path planning we have used a simulation approach. We assume a scenario which has two types of classes for the events; one is normal routine events while the other events are those that require an emergency response. To monitor these events a UWSN has been deployed. The nodes in the UWSN collect multimedia information through cameras. Due to a limited finite capacity, data needs to be offloaded from these nodes by an AUV. The nodes communicate infotential data using the acoustic communication medium. A path planning agent, based on the infotential data that it receives, schedules a visitation sequence of the nodes for the AUV. During its tour, the AUV off-loads multimedia data from the nodes using the optical communication medium. A node which has recorded an event that can be classified as an emergency is marked as a hot-spot.

The simulation has 100 nodes arranged in a 10×10 mesh/grid. The horizontal/vertical inter-node distance is 1000 m while the diagonal distance is 1414.2 m. The AUV traverses the UWSN at a constant speed of 10 m/s.

We assume that before the AUV embarks on its tour, the UWSN has been recording data for 24 h. Each node has video data reports of length 15 min each. Therefore, the reports have been recorded starting at intervals in the multiples of 15 min and with no recording overlap. The event coverage of the nodes is such that they have minimum or no-overlap, and hence, the recorded events are unique. Each node records data reports and classifies them either as a high-priority or a low-priority event. A {valuation,decay} tuple, corresponding to significance and damage rate, is appended to each data report. If it is a routine low-priority event, then the {valuation,decay} tuple $\left\{A_{L},B_{L}\right\}$ are appended to the data report. Alternatively, if it is an emergency event, then the tuple $\left\{A_{H},B_{H}\right\}$ are attached to it. The UWSN nodes communicate with the remote path-planning agent over the acoustic channel and transmit to it the {valuation,decay} and time-stamp details of the data reports that they have recorded. Based on these details, the path-planning agent determines the visitation sequence for the AUV.

### 5.3. Results

In this section, we attempt to make a comparative analysis of the path planning algorithms. We ascertain the performance of algorithms that take into consideration VoI or time or both, versus those algorithms, that do not. The regions where a high-priority event takes place are deemed as hot-spots. The effect of parameters such as valuation ratio $A_{H}/A_{L}$ and number of hot-spots $N_{HS}$ is assessed.

We make a performance comparison among six path planners; RPP, LPP, GPP, HPP, GIPP, and HIPP. At various points in this section, we normalize results. The normalization is with respect to RPP as it is our base-case. Except for in anomalous cases, RPP is the worst performing among all path planning algorithms; this is expected as it does not take into account time or information valuation for generating the node visitation sequence. Each reading in the results has been averaged over 100 different VoI profiles across the same UWSN map.

#### 5.3.1. Valuation Ratio

**Figure 3:**
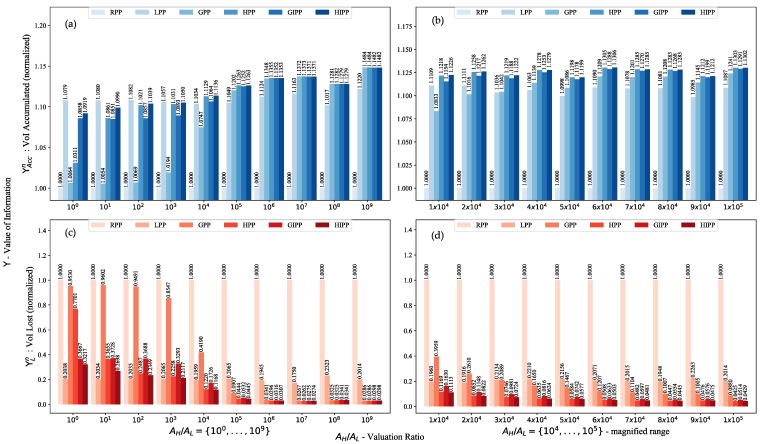
The top two graphs (colored in blue) are for $\Upsilon_{Acc}$ while the bottom two are for $\Upsilon_{L}$ while the graphs on the left are for the valuation ratio range $\left[10^{0}:10^{9}\right]$ while the graphs on the right are a magnification between $\left[10^{4}:10^{5}\right]$: (**a**) The top-left graph is for $\Upsilon_{Acc}^{n}$ which is charted in the range $\left[10^{0}:10^{9}\right]$; (**b**) The top-right graph is for $\Upsilon_{Acc}^{n}$ which is charted in the range $\left[10^{4}:10^{5}\right]$; (**c**) The bottom-left graph is for $\Upsilon_{L}^{n}$ which is charted in the range $\left[10^{0}:10^{9}\right]$; (**d**) The bottom-right graph is for $\Upsilon_{L}^{n}$ which is charted in the range $\left[10^{4}:10^{5}\right]$.

The first thing that we investigate is the valuation ratio. The valuation ratio is the ratio between the valuation of information of a high-priority event versus valuation of information of a low-priority event at $t=\tau_{o}$, i.e., the ratio $A_{H}/A_{L}$. In hindsight of the results for $N_{HS}=0$, we found that if the VoI profile in a UWSN is similar or homogeneous, i.e., all the sensor nodes have almost a similar amount of VoI to offer, then a shortest path algorithm proved quite effective. This effectiveness is a result of distance minimization which results in minimization of time, and this time minimization helps to lock-in decaying VoI profiles at the earliest. However, so should be the case if $A_{H}\approx A_{L}$, because, even though there are hot-spots, yet the VoI profile of the system is as if $N_{HS}=0$. In such a case, there would be no real advantage of using a greedy planner. Therefore, there should be a particular range after which we can observe a performance gain for the greedy planners as compared to the shortest path planners. In this experiment, we determine the effective performance range for the lawn-mower, greedy and hybrid algorithms. The results for $\Upsilon_{Acc}^{n}$ and $\Upsilon_{L}^{n}$ versus $A_{H}/A_{L}$ are shown in Figure 3. The superscript *n* implies that the results have been normalized with respect to RPP.

(29) $$\begin{array}{c} \Upsilon_{Acc}^{n}=\frac{\Upsilon_{Acc}^{PP}}{\Upsilon_{Acc}^{RPP}},\Upsilon_{L}^{n}=\frac{\Upsilon_{L}^{PP}}{\Upsilon_{L}^{RPP}} \end{array}$$

In Figure 3, the left two graphs are for the range $A_{H}/A_{L}=\left[10^{0}:10^{9}\right]$. The right two graphs are a magnification of the results between the range $A_{H}/A_{L}=\left[10^{4}:10^{5}\right]$. Also, the top two graphs are for $\Upsilon_{Acc}^{n}$ (colored in tones of blue), while the bottom two graphs are for $\Upsilon_{L}^{n}$ (colored in tones of red). We can observe that LPP performs better early on but once the ratio $A_{H}/A_{L}$ becomes considerably large, the greedy algorithms start giving better performance. It is somewhere between a valuation ratio of $10^{3}$ and $10^{4}$ units that LPP loses its top spot on performance. At $10^{4}$ GIPP, HPP, and HIPP start performing better. While at $10^{5}$ GPP also starts performing better than LPP. LPP clearly performs best up to $10^{3}$, while afterward, the greedy or hybrid algorithms start performing better. If we magnify the range between $A_{H}/A_{L}=\left[10^{4}:10^{5}\right]$, we find that the switch in performance takes place at $A_{H}/A_{L}=3\times 10^{4}$.

It is based on these results that we use a setting of $A_{H}/A_{L}=5\times 10^{4}$ for the rest of this paper. This is a reasonable number for practical situations as e.g., it may imply a $1 versus $50,000 valuation. However, it is to note that the hybrid or intermediate node visitation algorithms perform better as early as a fiscal value of $10,000; as suggested by the performance improvement at $A_{H}/A_{L}=10^{4}$.

#### 5.3.2. Justification of Heuristics

The algorithms proposed in this paper are based on different heuristics. In this section, we verify whether our intuitions behind those heuristics are valid or not. We use $\Upsilon_{ACC}^{n}$ (values normalized with respect to RPP) and study the performance of the path planners for the case where the number of hot-spots $N_{HS}=\left\{0,1,10\right\}$.

**Figure 4:**
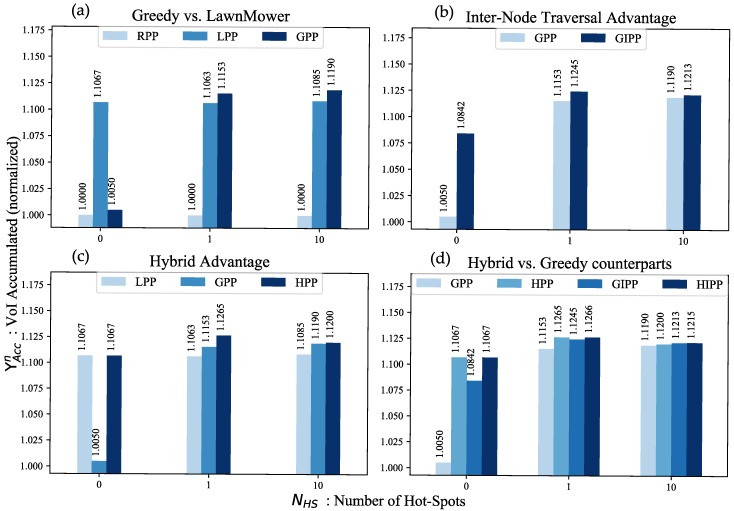
Bar graphs for justifying the use of various heuristics: (**a**) Greedy vs. Lawn-Mower; (**b**) Inter-Node Traversal Advantage; (**c**) Hybrid Advantage; (**d**) Hybrid vs. Greedy counterparts.

Our first hypothesis was that using VoI aware algorithms may help in accumulating a higher amount of VoI. From the results in Figure 4 we can see that all path planners have a value greater than 1.0, i.e., they are all better than RPP, and hence, suggesting that the hypothesis is correct.

Our second hypothesis was that greedy path planners amass a higher VoI when there are hot-spots. However, when there are no hot-spots, a time minimization planner like the Lawn-Mower accumulates a higher VoI. This hypothesis is validated through the results in Figure 4a. When $N_{HS}=0$ LPP performs best, but in the case of hot-spots, the greedy approach accumulates more VoI.

Our third hypothesis was that inter-node traversal helps in minimizing time while still maintaining its VoI greedy character, thereby, improving the VoI accumulated. GIPP, for instance, visits hot-spots on priority but also visits other nodes that lie along the path. Figure 4b confirms this intuition where GIPP accumulates more VoI than GPP.

Our fourth hypothesis was that hybrid path planners can provide the best of both worlds, i.e., when $N_{HS}=0$ they behave like time minimization path planners and when there are hot-spots they use greedy techniques for scheduling purposes (until all hot-spots have been visited). Figure 4c,d are a proof for this. When there are no hot-spots HPP performs as good as the LPP (because their algorithmic construction, in this case, is the same) and in case of hot-spots, they accumulate more VoI than their greedy counterparts, i.e., GPP and GIPP. Figure 4c shows that the hybrid algorithm is better than both LPP and GPP while Figure 4d shows that hybrid algorithms are better than their greedy counterparts in both cases, i.e., with or without intermediate node visitation.

#### 5.3.3. Comparative Analysis

In this section, we do a thorough comparative analysis to have a better understanding of the performance dynamics of the various path planners. The parameters we explore for this purpose are $\Upsilon_{ACC}$, $\Upsilon_{L}$, $T_{tour}$ & $\Omega^{n}$. We vary the number of hot-spots $N_{HS}$ from 0 to 10 and see the effect on the path planners’ performance.

**Figure 5:**
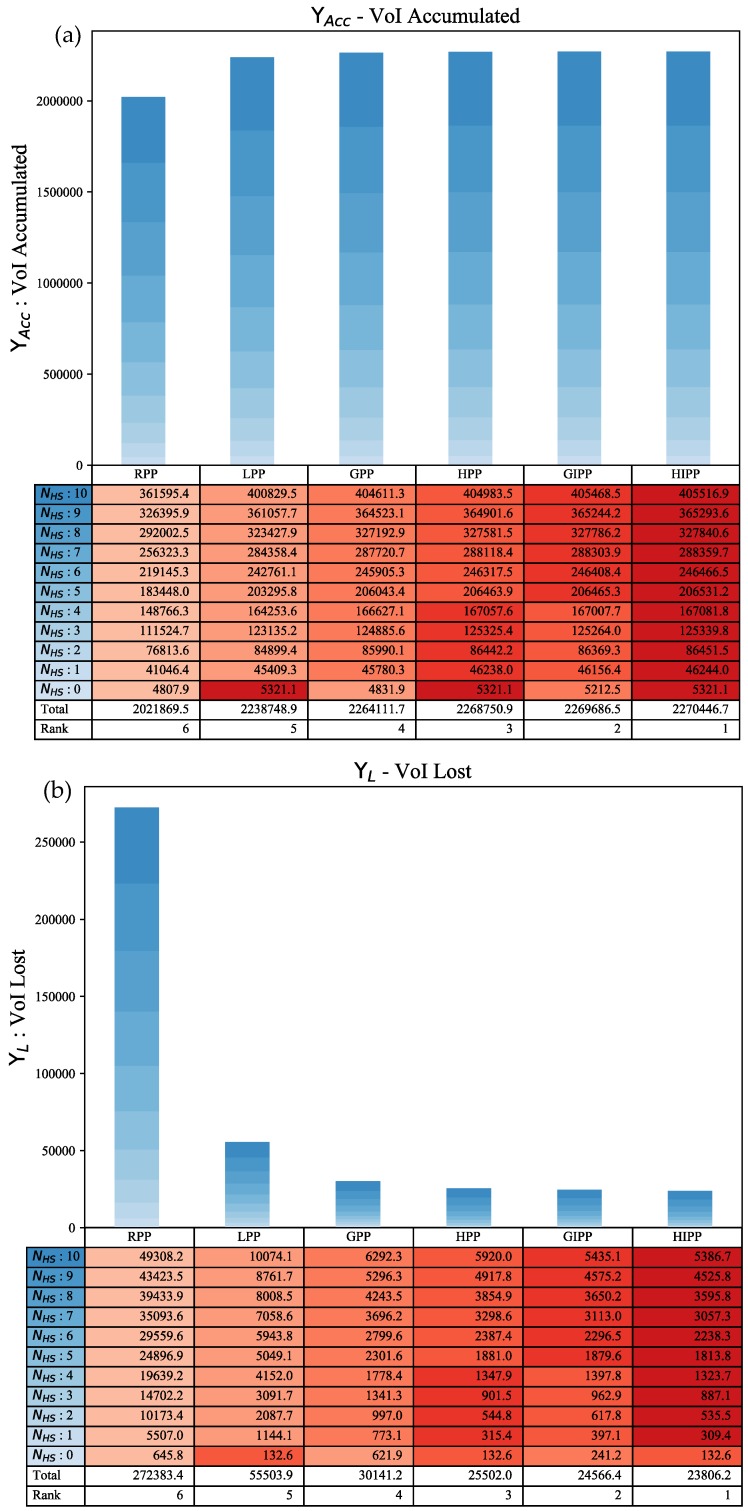
Comparative analysis for $\Upsilon_{ACC}$ & $\Upsilon_{L}$: (**a**) The top stacked bar graph is for VoI accumulated $\Upsilon_{ACC}$; (**b**) The bottom stacked bar graph is for VoI lost $\Upsilon_{L}$.

**Figure 6:**
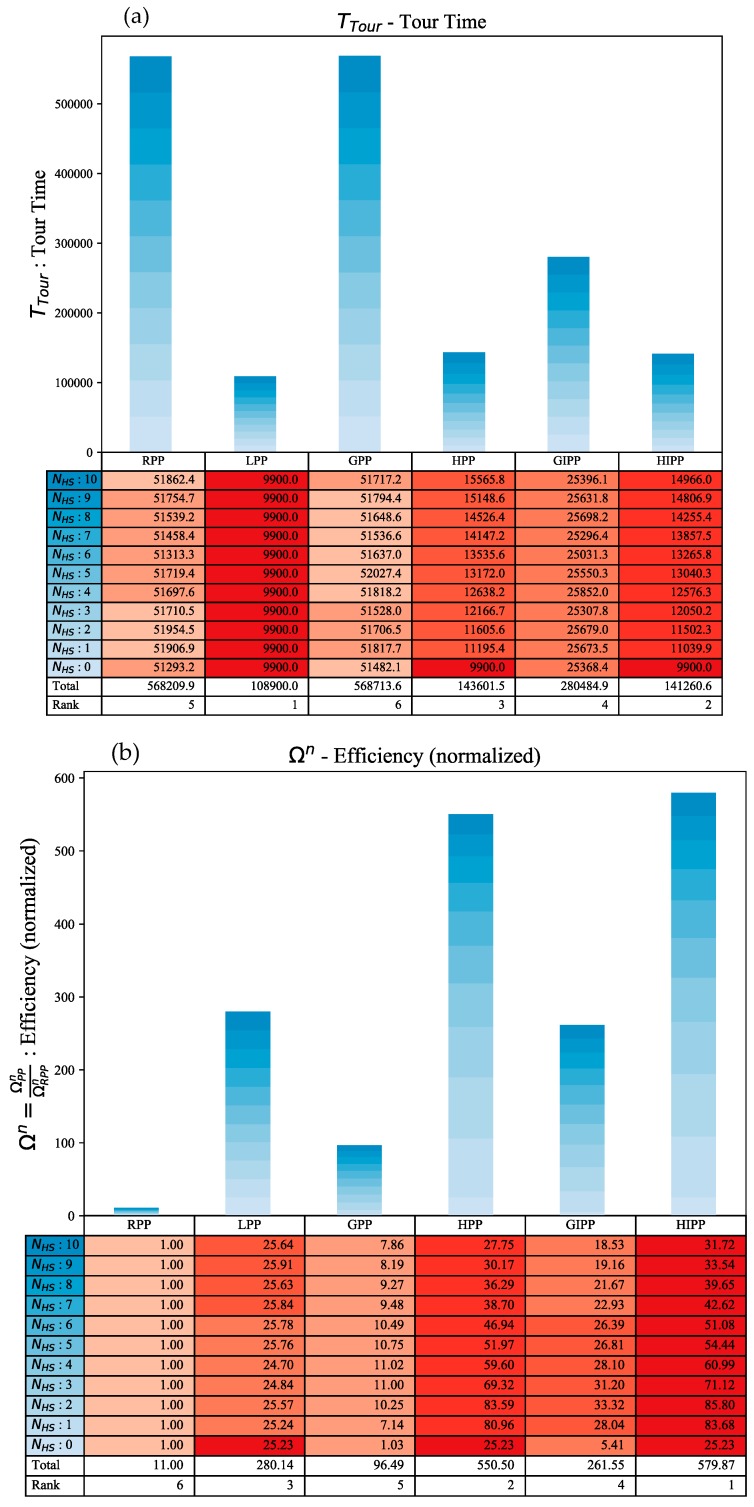
Comparative analysis for $T_{tour}$ & Ω: (**a**) The top stacked bar graph is for time to complete the tour $T_{tour}$; (**b**) The bottom stacked bar graph is for the efficiency measure Ω.

For this section we have used stacked bar-plots (Figure 5 and Figure 6). The results have not been normalized to give a more clear picture of the actual values obtained from the simulation. Each reading for a {Planner, Hot-Spot(s)} tuple is averaged over 100 different VoI profiles and is represented by a block in the stacked bar-plot. The exact value of a block is given in a corresponding cell in the table below. The changing color gradient of the blocks matches to the index column $N_{HS}$ in the table. The changing color tones of the table cells correspond to a change in intensity of values. The better the performance, the darker the color tone is in the table. Color tones for comparative performance can only be compared across a row, i.e., values can be only compared across a particular $N_{HS}$ value.

##### VoI Accumulated and VoI Lost

This discussion refers to graphs in Figure 5. The bar graph in Figure 5a is for VoI accumulated $\Upsilon_{ACC}$ by various path planners, while Figure 5b pertains to VoI lost $\Upsilon_{L}$. They have the same conclusions in terms of performance but the performance results for $\Upsilon_{L}$ are more pronounced as compared to $\Upsilon_{ACC}$ and this is because, as stated earlier, they remove the bias due to loss before $t=\tau_{Start}$. LPP performs best when there are no hot-spots. In this case, the performance of HPP and HIPP is same as LPP. With $N_{HS}>0$ the greedy and hybrid approaches start to perform better. The hybrid algorithms always perform better than their greedy counterparts. Also, GIPP always performs better than GPP. An interesting thing to note is that initially HPP has better performance than GIPP but after $N_{HS}=4$ the GIPP path planner starts outperforming HPP; which is due to the fact that the AUV using GIPP might start encountering lesser valued hot-spots more frequently while on its way towards a higher valued hot-spot; a benefit in hindsight of using intermediate node visitation. The cumulative performance rank for $N_{HS}=\left[0:10\right]$ is: HIPP > GIPP > HPP > GPP > LPP > RPP.

##### Tour Time

The best performing algorithm in terms of time is shortest path algorithm LPP and this can be seen from the results in Figure 6a. HIPP and HPP come in close in terms of minimizing time, but as the number of hot-spots increase, their performance gap to LPP also widens. GIPP performs better than GPP because of using the intermediate node visitation heuristic. GPP is as worse as RPP in terms of time, which is understandable as the VoI profile is distributed randomly across the UWSN map. It is important to note that when $N_{HS}=0$ then LPP = HPP = HIPP. The performance rank precedence across $N_{HS}=\left[0:10\right]$ is: LPP > HIPP > HPP > GIPP > GPP ≈ RPP.

##### Efficiency

This is a useful metric as it takes into account both the VoI lost and tour time. The bar graph for this is shown in Figure 6b. The hybrid algorithms turn out to be the best in terms of this metric. This is not a surprise as they combine the best of both the shortest path and greedy algorithms. The cumulative efficiency rank is: HIPP > HPP > LPP > GPP > GIPP > RPP.

#### 5.3.4. Scalability with an Increase in Hot-Spots

In the context of this discussion, a hot-spot is an anomalous event of probably catastrophic proportions and needs to be taken care of as quickly as possible. Such an anomalous event should be a rare occurrence and it would be a bit unlikely to find more than a few at the same time. However, then it can also be argued that the whole system is compromised if such a situation can arise. However, this scalability study is based on this hypothetical assumption that there might be a drastic increase in the number of hot-spots. Our goal is to see how the path planners behave when we vary the number of hot-spots ($N_{HS}$) across the range of nodes.

**Figure 7:**
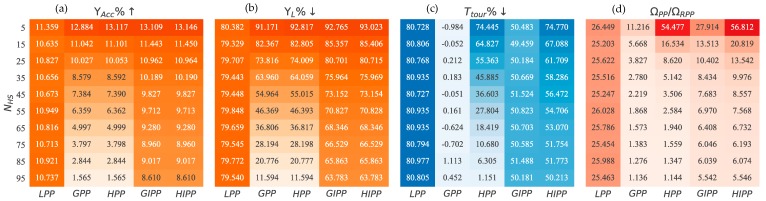
Scaling of response w.r.t. number of hot-spots $N_{HS}$: (**a**) Percentage improvement in VoI accumulated $\Upsilon_{ACC}$; (**b**) Percentage reduction in VoI lost $\Upsilon_{L}$; (**c**) Percentage reduction in tour time $T_{Tour}$; (**d**) Normalized measure of efficiency Ω.

The results are shown in Figure 7. The results are shown as heat-maps. Darker color tones imply better comparative performance in the heat-maps. We start from 5 hot-spots and ramp up to 95 hot-spots. We record percentage improvement in VoI accumulated ($\Upsilon_{ACC}\%↑$), percentage reduction in VoI lost ($\Upsilon_{L}\%↓$), percentage reduction in tour time ($T_{Tour}\%↓$) and a normalized measure of efficiency ($\frac{\Omega_{PP}}{\Omega_{RPP}}$). The percentages are calculated with respect to the RPP path planner performance:

(30) $$\begin{array}{c} \Upsilon_{ACC}\%↑=\frac{\Upsilon_{ACC}}{\Upsilon_{ACC}^{RPP}}\times 100 \end{array}$$

(31) $$\begin{array}{c} \Upsilon_{L}\%↓=\frac{\Upsilon_{L}}{\Upsilon_{L}^{RPP}}\times 100 \end{array}$$

(32) $$\begin{array}{c} T_{Tour}\%↓=\frac{T_{Tour}}{T_{Tour}^{RPP}}\times 100 \end{array}$$

(33) $$\begin{array}{c} \frac{\Omega_{PP}}{\Omega_{RPP}}=\frac{\Upsilon_{L}^{RPP}\times T_{Tour}^{RPP}}{\Upsilon_{L}\times T_{Tour}} \end{array}$$

The first results we discuss are $\Upsilon_{ACC}$ and $\Upsilon_{L}$. As the number of hot-spots increase, the performance of HPP and LPP decreases drastically. GIPP and HIPP also experience a degradation in performance but the change is not that drastic. The performance of LPP remains the same at average. The reason for this is that an increase in hot-spots results in a map that is increasingly homogeneous in terms of VoI. When there are no hot-spots, there is a homogeneity in terms of VoI in the sensor map. In such a case, as discussed earlier, LPP performs best. With one and up till a few hot-spots, greedy path-planners perform better. However, at a certain tipping point in terms of $N_{HS}$, the performance falls below than that of LPP. With a large $N_{HS}$, the sensor map is similar to one with no hot-spots. This explains the better performance of LPP in such a scenario.

In terms of $T_{tour}$, there is no effect in the performance of LPP, GPP or GIPP with an increase in $N_{HS}$. However, the performance of HPP deteriorates to the level of GPP eventually and HIPP degrades to GIPP. The reason lies in the algorithmic construction of the hybrid path planners. They are designed in such a way that they switch to LPP once all the hot-spots have been visited. The time performance of hybrid algorithms is good because they incorporate LPP. Therefore, if there is a large $N_{HS}$, there will be a lesser involvement of LPP in the path-planning process and this leads to an increase in the $T_{tour}$ for hybrid path-planners.

The measure of efficiency, normalized to RPP, has a very high value for hybrid algorithms at 5 hot-spots. However, onward 15 hot-spots, we see a very sharp decline in this performance measure. LPP is not affected in this regard. The reason is that Ω depends upon $\Upsilon_{L}$ and $T_{tour}$ and LPP is not affected in both regards by an increase in $N_{HS}$.

#### 5.3.5. Response to Emergency

**Figure 8:**
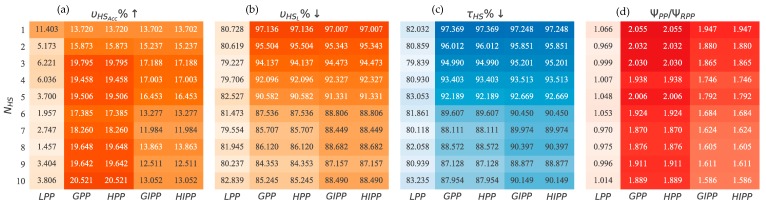
Emergency Response w.r.t. number of hot-spots $N_{HS}$: (**a**) Percentage improvement in VoI accumulated from first hot-spot $\upsilon_{HS_{ACC}}$; (**b**) Percentage reduction in VoI lost from first hot-spot $\upsilon_{HS_{L}}$; (**c**) Percentage reduction in time to reach first hot-spot $\tau_{HS}$; (**d**) Normalized urgency score Ψ.

The basic reason to employ VoI was to have a mechanism to distinguish between higher and lower priority situations in an organic fashion, thus enabling a more appropriate response to the situation. The greedy and hybrid algorithms were designed to address high-priority situations such as emergencies. Here, we look at some measures that shed light on how the various path planners perform under emergency. Again, all results are described with respect to RPP in terms of percentage improvement in performance. The results are shown in Figure 8. We vary $N_{HS}$ from 1 to 10 hot-spots. We record percentage improvement in VoI accumulated from first hot-spot ($\upsilon_{HS_{ACC}}\%↑$), percentage reduction in VoI lost at first hot-spot ($\upsilon_{HS_{L}}\%↓$), percentage reduction in tour time to first hot-spot ($\tau_{HS}\%↓$) and the normalized urgency score ($\frac{\Psi_{PP}}{\Psi_{RPP}}$). The percentages are calculated with respect to the RPP path planner performance:

(34) $$\begin{array}{c} \upsilon_{HS_{ACC}}\%↑=\frac{\upsilon_{HS_{ACC}}}{\upsilon_{HS_{ACC}}^{RPP}}\times 100 \end{array}$$

(35) $$\begin{array}{c} \upsilon_{HS_{L}}\%↓=\frac{\upsilon_{HS_{L}}}{\upsilon_{HS_{L}}^{RPP}}\times 100 \end{array}$$

(36) $$\begin{array}{c} \tau_{HS}\%↓=\frac{\tau_{HS}}{\tau_{HS}^{RPP}}\times 100 \end{array}$$

(37) $$\begin{array}{c} \frac{\Psi_{PP}}{\Psi_{RPP}}=\frac{S_{I}}{S_{P}}\times \frac{S_{P}^{RPP}}{S_{I}^{RPP}} \end{array}$$

GPP and HPP are best when it comes to accumulating VoI from the first hot-spot. GIPP and HIPP follow closely in terms of performance but the gap widens with increasing $N_{HS}$. The reason, as discussed earlier, is that the intermediate path planners start to hit lesser valued hot-spots on their way to the highest valued hot-spot. As an intermediate lesser valued hot-spot maybe encountered first by the AUV, therefore, $\upsilon_{HS_{ACC}}$ performance should decrease.

In terms of $\upsilon_{HS_{L}}$, GPP and HPP are better for $N_{HS}$ = 1 or 2. For $N_{HS}\geq 3$, GIPP and HIPP start performing better, i.e., they avoid a higher loss in terms of VoI from the first hot-spot encountered. The reason is the same as stated above, i.e., they encounter other hot-spots on the tour while traveling towards the highest-priority one. Because, hot-spots are encountered earlier, therefore, the respective VoI loss $\upsilon_{HS_{L}}$ at that node should be lower. This phenomenon of encountering hot-spots earlier than planned can be verified from the time to arrive at the first hot-spot $\tau_{HS}$ results.

These results for $\tau_{HS}$ follow exactly $\upsilon_{HS_{L}}$ in character. Again, GPP and HPP perform better for $N_{HS}$ = 1 or 2, while for $N_{HS}\geq 3$, GIPP and HIPP are better. This thus corroborates the speculation that hot-spots are being encountered earlier by path planners that are based on the intermediate node visitation strategy.

The urgency measure Ψ sheds light on response to an emergency. This is because it generates a score based on the sequence of visitation to the hot-spots. It encodes, that how much priority was maintained while visiting the nodes. GPP and HPP have the highest urgency score. This is because they visit the nodes in the exact order of descending priority as dictated by VoI advertised. The intermediate node visitation algorithms, GIPP and HIPP, come in second. This is expected as intermediate nodes are being attended to en-route to the highest-priority node. The performance gap widens between algorithms with or without intermediate node visitation with an increasing $N_{HS}$. The performance of LPP is as worse as RPP throughout the $N_{HS}$ range. This is inferred from normalized result value of $\Psi_{LPP}/\Psi_{RPP}\approx 1$.

This shows that GPP and HPP are best for addressing emergencies as they directly go to the highest priority node first. Close in second are GIPP and HIPP. They lose out marginally because of visiting intermediate nodes. LPP has no capacity for dealing with emergencies.

## 6. Conclusions

In this paper, we have proposed the use of VoI in the form of infotentials for solving data off-loading precedence issues in UWSNs. We have proposed a VoI model for UWSNs and developed various measures and metrics to asses system performance. A relationship has already been identified between the quality of information QoI and value of information VoI in [13,15]. Based on this relationship and the extensive experiments that we have performed, it is reasonable to conclude that employing VoI for path planning algorithms improves the quality of information being retrieved from a UWSN.

The path planner performance depends on the context of the situation. If the VoI profile in the system is homogeneous, i.e., there are no hot-spots or the valuation ratio $A_{H}/A_{L}$ is small, then a shortest path algorithm like LPP should be used. LPP is also fuel optimal. In the case of hot-spots, given the valuation ratio $A_{H}/A_{L}$ is considerably large, greedy algorithms perform better. Hybrid algorithms offer the best of both these strategies by combining greedy and shortest path algorithms.

Intermediate node visitation improves VoI by saving tour time and, therefore, GIPP and HIPP perform better than their GPP and HPP counterparts respectively. However, if an emergency is classified as so serious that its priority should not be marginalized, then GPP or HPP should be used as they directly visit the node of concern.
